# When is the Rat Retrosplenial Cortex Required for Stimulus Integration?

**DOI:** 10.1037/bne0000267

**Published:** 2018-10

**Authors:** Andrew J. D. Nelson, Emma L. Hindley, Seralynne D. Vann, John P. Aggleton

**Affiliations:** 1 School of Psychology, Cardiff University

**Keywords:** cingulate cortex, configural learning, conditioned inhibition, nonspatial memory

## Abstract

The rodent retrosplenial cortex is known to be vital for spatial cognition, but evidence has also pointed to a role in processing nonspatial information. It has been suggested that the retrosplenial cortex may serve as a site of integration of incoming sensory information. To examine this proposal, the current set of experiments assessed the impact of excitotoxic lesions in the retrosplenial cortex on two behavioral tasks that tax animals’ ability to process multiple and overlapping environmental stimuli. In Experiment 1, rats with retrosplenial lesions acquired a negative patterning discrimination, a form of configural learning that can be solved only by learning the conjunction of cues. Subsequent transfer tests confirmed that both the lesion and control animals had solved the task by using configural representations. Furthermore, in Experiment 2, a 2nd cohort of retrosplenial lesion animals successfully acquired conditioned inhibition. Nevertheless, the same animals failed a subsequent summation test that assesses the ability to transfer what has been learned about one stimulus to another stimulus in the absence of reinforcement. Taken together, these results suggest that in the nonspatial domain, the retrosplenial cortex is not required for forming associations between multiple or overlapping environmental stimuli and, consequently, retrosplenial engagement in such processes is more selective than was previously envisaged.

Given the dense interconnections of the rodent’s retrosplenial cortex (RSC; areas 29, 30) with the hippocampus and anterior thalamic nuclei, research into its functions has understandably focused on its role in spatial learning and memory (Miller, Vedder, Law, & Smith, 2014; Vann, Aggleton, & Maguire, 2009). However, a consideration of the other connections of the retrosplenial cortex points to an additional role in processing nonspatial information. For example, the retrosplenial cortex receives visual information directly from the geniculostriate and tecto-cortical visual systems and shares reciprocal connections with the parietal and parahippocampal cortices (Van Groen & Wyss, 1990, 1992a, 1992b; Van Groen & Wyss, 2003). Indeed, research has shown that the rat retrosplenial cortex contributes to learning about stimuli that are not explicitly spatial (Bussey, Muir, Everitt, & Robbins, 1996; Hindley, Nelson, Aggleton, & Vann, 2014; Jiang, DeAngeli, Bucci, & Todd, 2018; Keene & Bucci, 2008a, 2008b; Kwapis, Jarome, Lee, & Helmstetter, 2015; Miller et al., 2014; Nelson, Hindley, Haddon, Vann, & Aggleton, 2014; Robinson, Keene, Iaccarino, Duan, & Bucci, 2011; Robinson et al., 2014; Smith, Freeman, Nicholson, & Gabriel, 2002; Smith, Miller, & Vedder, 2018).

A core feature of many of these tasks is the formation of interrelationships among multiple, and often competing or overlapping, stimuli. However, the picture is complicated by findings that the retrosplenial cortex is not required for all forms of stimulus−stimulus learning (e.g., Todd, Huszár, DeAngeli, & Bucci, 2016) as well as the fact that some of the evidence has been derived from studies employing electrolytic lesions (e.g., Keene & Bucci, 2008a; Robinson et al., 2011). This surgical method creates the risk of unintended white matter damage. Thus, the specific contribution of the retrosplenial cortex to stimulus processing has not been established. One possibility is that the retrosplenial cortex is required for the integration of different classes of sensory information that in turn support the formation of contextual and spatial representations (Todd & Bucci, 2015; Todd, DeAngeli, Jiang, & Bucci, 2017). Alternatively, the retrosplenial cortex may have a more selective role in stimulus processing and serve as a comparator that translates representations from one frame of reference to another that may include in the nonspatial domain the updating of representations when stimulus relationships change (Burgess, 2002; Byrne, Becker, & Burgess, 2007; Clark, Simmons, Berkowitz, & Wilber, 2018; Vann et al., 2009). The current experiments sought to contrast these two theoretical accounts of retrosplenial cortex function.

Configural learning arguably represents the archetypal test of stimulus integration. Tests of configural learning can be solved only by learning the conjunction of cues (configural) rather than single cues (elements) because they involve discriminating between different combinations of common elements. Configural learning is also of interest because the ability to combine information from multiple stimuli is thought to be key for some forms of spatial navigation (O’Keefe & Nadel, 1978), where the combinations of cues available from any particular location will differ but are likely to overlap with the cues visible in other nearby locations. Given the known involvement of the retrosplenial cortex in processing allocentric information (Czajkowski et al., 2014; Nelson, Powell, Holmes, Vann, & Aggleton, 2015; Vann & Aggleton, 2002), configural processing may be a core retrosplenial function underlying its role in both spatial and nonspatial cognition. To examine a potential role of the retrosplenial cortex in configural learning, we tested animals with excitotoxic lesions in the retrosplenial cortex on a negative patterning task (Experiment 1). Negative patterning requires animals to learn a patterning discrimination in which rewards are delivered after either of two stimuli are presented individually but not when the stimuli are presented in compound (A+, B+, AB−; see Figure 1). Subsequent transfer tests examined whether the rats solved the negative patterning task using nonconfigural strategies such as numerosity of cues (Bussey et al., 2000).[Fig-anchor fig1]

Experiment 2 further examined retrosplenial involvement in stimulus processing by assessing the impact of retrosplenial damage on a conditioned inhibition discrimination. Conditioned inhibition is not configural in nature but nonetheless requires animals to learn interrelationships between competing cues because the presence of one stimulus signals that another stimulus will not be followed by reinforcement. Although there have been two previous reports of disrupted conditioned inhibition performance after retrosplenial damage (Keene & Bucci, 2008a; Robinson et al., 2011), the current approach differed in two important aspects. First, we employed axon-sparing excitotoxic lesions that potentially allow for a more selective assessment of retrosplenial function (Aggleton & Vann, 2004; Meunier & Destrade, 1988, 1997). Second, successful acquisition of conditioned inhibition was confirmed by administration of both retardation and summation tests (Cole, Barnet, & Miller, 1997; Rescorla, 1969). The summation test, which involves pairing the putative conditioned inhibitor with a novel excitatory conditioned stimulus (conditioned stimulus [CS]), is of particular interest because it tests the ability to transfer what has been learned about one stimulus to another stimulus in the absence of reinforcement (see Figure 1). Thus, if the role of the retrosplenial cortex in stimulus processing is a more selective translational one, then animals with lesions in the retrosplenial cortex may be able to acquire conditioned inhibition (integrate) but fail the summation test when this information needs to be generalized to a novel stimulus (translation).

## Method

### Subjects and Materials

The two cohorts (RSC1 and RSC2) comprised 56 experimentally naïve, male Lister Hooded rats (Harlan, Bicester, United Kingdom). The rats at the time of surgery in RSC1 cohort weighed 278−387 g, and the rats in RSC2 weighed 244–296 g. The rats were housed in pairs in a temperature-controlled room. Lighting was kept on a 12-hr light−dark cycle, light from 0800 to 2000. Water was available ad libitum throughout the experiments. For all behavioral experiments, the animals were placed on a food-restricted diet where they were able to gain weight. Their weights did not fall below 85% of their free-feeding weights. All experiments were carried out in accordance with U.K. Animals (Scientific Procedures) Act 1986 and EU directive (2010/63/EU). Rats were provided with cardboard tubes and wooden chew sticks in their home cages. Animals in each cohort received either a bilateral excitotoxic lesion (RSC1 *n* = 16; RSC2 *n* = 16) or a sham lesion (Sham1 *n* = 12; Sham2 *n* = 12) of areas 29 and 30.

### Surgical Procedure

Rats were deeply anesthetized with an intraperitoneal (i.p.) injection of sodium pentobarbital (60 mg/kg pentobarbital sodium salt; Centravet, Dinan, France). Oxygen was provided throughout the surgery. If required, animals were given an additional .03-ml injection of sodium pentobarbital to maintain anesthesia. If further anesthesia was still required, <1% inhaled isoflurane was given. All animals received a subcutaneous injection of .06 ml Metacam (Boehringer Ingelheim, Alkmaar, The Netherlands) for postoperative analgesia. The scalp was shaved, and the animal was placed in a stereotaxic frame (David Kopf Instruments, Tujunga, CA) with the nose bar set at +.5. The skull was exposed, and a bilateral craniotomy extending from bregma to lambda was made in the skull using a dental drill. The more posterior areas of the retrosplenial cortex were revealed by drilling away two short strips of skull from the opened area, leaving a strip of bone approximately 2 mm wide over the central sinus as protection.

Lesions were made by injecting .09 M N-methyl-D-aspartate (NMDA; Sigma, Poole, United Kingdom) dissolved in phosphate buffer (pH 7.2) into 14 injection sites at a rate of .05 μl per minute using a 1-μl Hamilton syringe (gauge 25s; Bonaduz, Switzerland). The stereotaxic coordinates of the lesion placements are stated relative to bregma in the anterior−posterior (AP) axis, and relative to the central sinus in the lateral−medial (LM) axis. Dorsal−ventral (DV) coordinates were taken relative to the surface of the cortex, using the eye of the needle.

The coordinates and injection volume of NDMA at each site for the RSC1 cohort were AP −1.6, LM ±.4, DV −1.3 (.25 μl); AP −2.8, LM ±.5, DV −1.3 (.25 μl); AP −4.0, LM ±.5, DV −1.3 (.25 μl); AP −5.3, LM ±.5, DV −2.6 (.26 μl); AP −5.3, LM ±.9, DV −1.6 (.26 μl); AP −6.6, LM ±1.0, DV −2.0 (.26 μl); and AP −7.5, LM ±1.1, DV −1.3 (.1 μl). For the RSC2 cohort the coordinates and NDMA injection volumes at each site were AP −1.8, LM ±.4, DV −1.0 (.25 μl); AP −2.8, LM ±.4, DV −1.1 (.26 μl); AP −4.0, LM ±.4, DV −1.1 (.26 μl); AP −5.3, LM ±.4, DV −2.4 (.3 μl); AP −5.3, LM ±.9, DV −1.4 (.29 μl); AP −6.6, LM ±.9, DV −1.8 (.29 μl); and AP −7.5, LM ±1.0, DV −1.0, (.1 μl).

After each infusion, the needle was left in situ for 5 min to allow absorption of the bolus. Following surgery, the scalp was sutured and a subcutaneous injection of 5 ml glucose−saline was given to replace lost fluids. Lidocaine (Xylocaine, AstraZeneca, United Kingdom) and antibiotic powder (Dalacin C, Pharmacia, United Kingdom) were applied topically to the wound, and animals were left to recover in a warm quiet area before being returned to their home cage. Sham animals underwent the same procedure, except that the needle was not lowered and no injections of neurotoxin were made. Postoperative care was identical for all groups. All animals recovered well following surgery.

### Histological Procedure

At the completion of all experiments, rats were deeply anesthetized using sodium pentobarbital (60 mg/kg, i.p.; Euthatal; Merial Animal Health, Harlow, United Kingdom), then transcardially perfused with .1 M phosphate-buffered saline (PBS) followed by 4% paraformaldehyde in .1 M PBS (PFA). The brains were removed and placed in PFA for 4 hr before being transferred to 25% sucrose and left overnight at room temperature, with gentle agitation. Four adjacent series of coronal sections (40 μm) were cut on a freezing sliding microtome. One series was mounted directly onto gelatin-coated slides after slicing and was stained using cresyl violet, a Nissl stain, for verification of the specific brain regions.

### Apparatus

Eight operant boxes measuring 30 cm wide × 24 cm deep × 21 cm high (Med Associates, George, VT) were used. Each chamber had three aluminum walls, and the fourth wall was composed of a Perspex door. The floor of the chamber was made up of 19 stainless steel rods, 1.6 cm apart. Each rod was 3.8 mm in diameter. Beneath the rods was a removable metal tray. Illumination in each chamber could be provided by a 3W house light located at the top center of the left-hand wall. In the center of the right-hand wall, a recessed magazine (5 cm tall × 5 cm wide) was located, via which Noyes food pellets (45 mg; Noyes, Lancaster, NH) could be delivered. The magazine could be illuminated by a light located within the ceiling of the recess. Above the magazine was a circular panel light 2 cm in diameter. To either side of the magazine was a flat-panel retractable lever, with a circular panel light 2 cm in diameter located above each lever. Auditory stimuli, delivered via speakers in the ceiling, consisted of a 2-kHZ tone or a series of clicks at 10 Hz. Visual stimuli consisted of two panel lights flashing (.1 s on, .1 s off) or the steady simultaneous illumination of both panel lights. Additional visual stimuli were provided by the magazine light and the central panel light. The same apparatus was used in both experiments.

### Behavioral Procedures

#### Experiment 1: Retrosplenial cortex and negative patterning

##### Negative patterning training

Initially, rats were trained to lever-press, so that after four training sessions each rat would lever-press for a single food pellet on a random interval schedule (RI15) such that a reward became available on average once in a 15-s period and the next lever-press response led to delivery of a reward. At the beginning of each session, the house light was illuminated and one of the levers was retracted. Each session lasted approximately 20 min.

Discrimination training commenced the next day. First, the rats learned that lever-pressing was reinforced during the presentation of two stimuli. The stimuli were either a tone (A+) or flashing panel lights (B+). Each of these stimuli was presented 12 times, with a varying interstimulus interval (mean = 60 s). The order of these trial presentations was pseudorandom, with the constraint that no more than two trials of the same type could occur consecutively. During the interstimulus interval, the lever was retracted, then extended again at the start of the subsequent trial. Each stimulus presentation lasted for 60 s. To assess animals’ learning of the discrimination uncontaminated by reinforcement, reinforcement was unavailable during the first 10 s of each trial. During the remaining 50 s, reinforcement was available, and consequently this provided a measure of both learning and performance. During the 50-s period, correct lever-pressing was reinforced according to the RI15 schedule of reinforcement. Training on A+ B+ lasted for 4 days.

Once responding to A+ and B+ had been established, rats received trials in which the tone and flashing light were presented simultaneously. Lever-pressing was not reinforced during presentations of the compound stimuli (AB−). In each training session, AB− was presented 12 times, whereas A+ and B+ were presented six times each. The order of these trial presentations was pseudorandom, with the constraint that no more than two trials of the same type could occur consecutively. Rats were trained on this negative patterning discrimination for 24 sessions.

##### Transfer tests

Following acquisition of negative patterning (A+ B+ AB−), two transfer tests were conducted. First, animals received three sessions of training with a novel rewarded stimulus: illumination of the magazine light (C+). The stimulus was presented 24 times in each session, and the same interstimulus intervals and stimulus duration were used as during the negative patterning training. Once lever-pressing to C+ had stabilized, C+ was introduced into the original negative patterning problem, giving A+ B+ AB− C+. The compound AB− was presented 12 times in each session, whereas the three elemental (rewarded) stimuli were presented four times each. Rats were given four sessions of training on this discrimination.

Training on A+ B+ AB− C+ was followed by the first transfer test, in which C+ was presented in combination with the tone or the flashing panel lights, that is, the original A+ and B+ stimuli. This tested whether animals had learned the negative patterning discrimination based on the number of stimuli presented simultaneously, instead of creating a configural representation of the AB− compound. If this were the case, responding to other stimulus compounds comprising two stimuli would be expected to be lower relative to responding to the elemental stimuli. Consequently, animals received trials in which C was paired with each elemental stimulus. If, however, a configural strategy had been used, responding to AC− and BC− would be expected to be higher than to AB−. The session consisted of four trials of each of the compound (AB−, AC−, BC−) and elemental (A+, B+, C+) stimuli.

Finally, to demonstrate that rats are able to suppress responding to compounds containing the magazine light, a final transfer test was carried out in which C+ was combined with AB− to produce an unrewarded compound: ABC−. During this session, the three original simple stimuli were each presented four times, and the two compounds AB− and ABC− presented six times each. Because ABC− contains the previously trained, unrewarded stimulus AB−, responding to the ABC− compound was expected to be low, showing that rats can suppress lever-pressing in the presence of the magazine light.

#### Experiment 2: Retrosplenial cortex and conditioned inhibition

A new cohort of rats (RSC2, Sham2) was used for these experiments.

##### Acquisition of conditioned inhibition

The rats initially learned to retrieve food pellets from the magazine well in a single 30-min session during which two pellets were delivered on a variable-time 60-s schedule. The house light was illuminated throughout the session.

Animals were then trained on an appetitive Pavlovian procedure to acquire conditioned inhibition to a stimulus termed *X.* The conditioned stimuli (CS) were 10 s in length. The CSs were presented in pseudorandom order, with an intertrial interval of 2 min (range = 1−3 min). Where appropriate, two food pellets were delivered at the end of CS presentation. The number of nose pokes made into the magazine well served as the measure of conditioned responding. To take account of any baseline differences in activity, a pre-CS measure was taken (magazine activity in a 10-s period before CS onset), and this was subtracted from the number of responses made during CS presentation. The three stimuli used were termed A (tone), B (central panel light), and X (house light off). Animals were first given four sessions in which there were six presentations of the rewarded stimulus A+ and six presentations of the unrewarded compound stimulus AX−. For the remaining eight sessions, there were an additional six presentations per session of the rewarded B+ stimulus. The house light remained illuminated throughout these sessions except when extinguished during presentations of X. The order of these trial presentations was pseudorandom, with the constraint that no more than two trials of the same type could occur consecutively.

##### Summation test

The ability of the conditioned inhibitor X to act as an inhibitor when presented in compound with the conditioned excitor B (with which it had not previously been paired) was then assessed in a summation test (Rescorla, 1969). In a single extinction session, conditioned responding was assessed to B and the compound BX. There were four presentations of each of the two stimuli (B, BX) with an intertrial interval of 2 min.

##### Retardation test

The retardation test assesses the ability of the previously trained conditioned inhibitor to acquire excitatory properties. Conditioned inhibition usually leads to retarded acquisition of excitatory conditioning relative to a novel stimulus that has not previously had inhibitory properties (Rescorla, 1969).

Following completion of the summation test, animals underwent six further sessions of training on the original conditioned inhibition discrimination. In each session, there were six presentation of A+ and six of AX−. In the final two sessions, the rats also received presentations of a novel unrewarded stimulus Y− (two presentations per session). Stimulus presentation was again pseudorandom, with the constraint that no more than two trials of the same type could occur consecutively. The small number of presentations of Y (magazine light) ensured that in the subsequent retardation test the stimulus had not received sufficient exposure to either acquire inhibitory properties or be subject to latent inhibition.

The subsequent retardation test assessed the strength of X as a conditioned inhibitor by comparing its ability to acquire excitatory properties with that of the neutral stimulus Y. Rats received three sessions with six presentations of each stimulus (X+ and Y+) per session. As before, stimulus presentation was pseudorandom, CS duration was 10 s, and two food pellets were delivered immediately on cessation of stimulus presentation.

### Data Analysis

**Figure 2 fig2:**
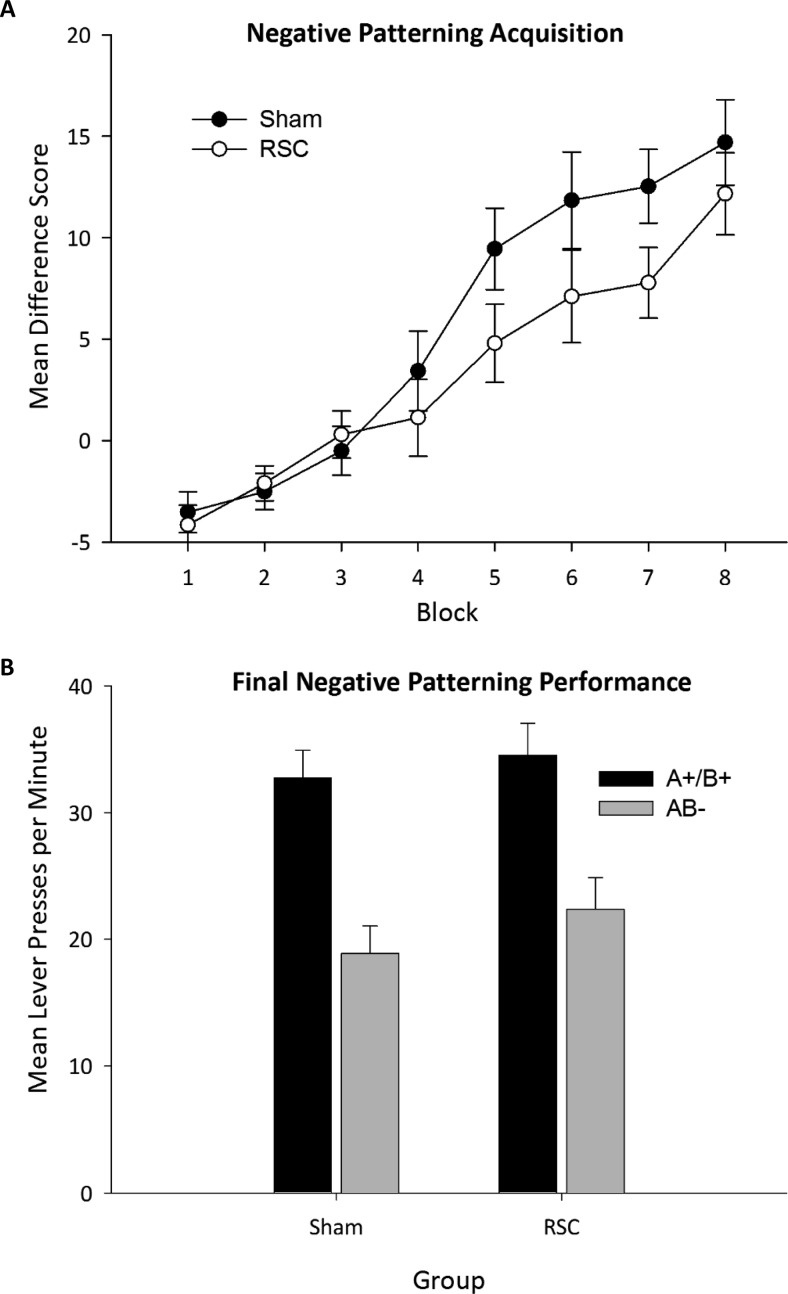
Panel A: Acquisition of the negative patterning discrimination for Sham1 and RSC1 lesion animals in blocks of three sessions (Experiment 1). The figure depicts a difference score calculated by subtracting the mean lever presses per minute during presentation of the reinforced tone (A+) and flashing panel light (B+) stimuli from the mean lever presses during the nonreinforced compound presentation of these stimuli (AB−). A number higher than 0 represents greater responding during reinforced (i.e., A+ and B+ trials) relative to nonreinforced (i.e., AB−) trials. Error bars represent the standard error of the mean. Panel B: Final performance levels of the Sham1 and RSC1 lesion animals on the negative patterning task (Experiment 1). Data are from the final 24-trial session. RSC = retrosplenial cortex lesion.

All data were analyzed by mixed analysis of variance (ANOVA) with within-subject factors of session (where appropriate) and stimulus, as well as a between-subjects factor of lesion. To verify that the assumptions of ANOVA were met, Levene’s test of equality of variances was conducted for all between-subjects comparisons and Mauchly’s test of sphericity for all analyses involving within-subject comparisons. Where violations of sphericity occurred, degrees of freedom were adjusted using the Greenhouse-Geisser correction. To examine within-subject differences in response rates to the different stimuli, pairwise comparisons (Bonferroni-corrected) were computed. In Experiment 1, the mean rate of lever pressing during the first 10 s of stimulus presentation (i.e., unrewarded period) served as the dependent variable because this provided a measure of discrimination learning uncontaminated by reward delivery. To assess any differences in performance as well as learning, lever-press behavior during the 50-s period in which correct lever presses were rewarded was also analyzed. In Experiment 2, the dependent variable was the number of magazine entries during CS presentation minus the number of magazine entries during the 10-s pre-CS epoch. The alpha level was set at *p* < .05. The error bars in Figure 2A represent the standard error of the mean. To control for between-subjects variability and construct error bars appropriate for within-subject comparisons (see Figures 2B and 4B−6B), the standard error of the mean was calculated using the method proposed by Cousineau (Cousineau, 2005; O’Brien & Cousineau, 2014). These error terms were then corrected according to the method proposed by Morey (Morey, 2008).[Fig-anchor fig2]

## Results

### Histological Evaluation of the Lesions

**Figure 3 fig3:**
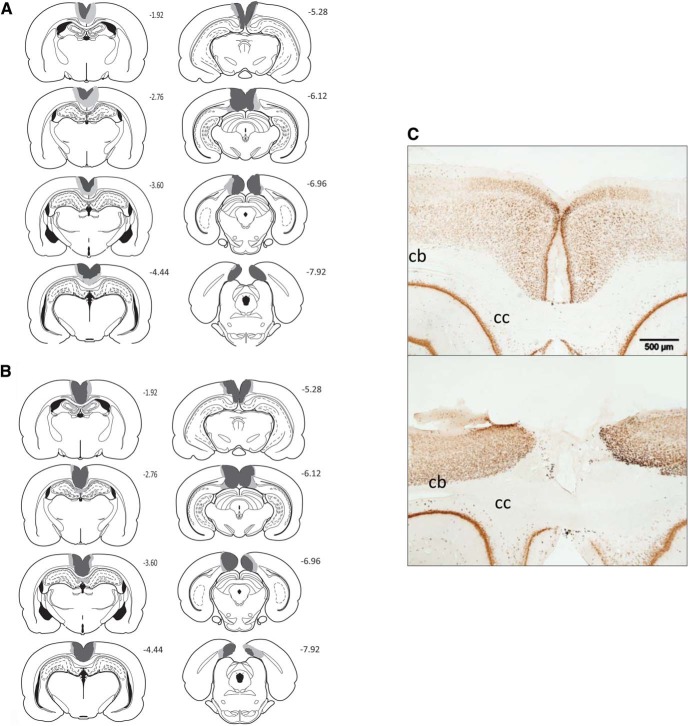
Location and extent of retrosplenial cortex (RSC) lesions. The coronal reconstructions show the cases with the minimal (dark gray areas) and maximal (light and dark gray areas) extent of retrosplenial damage in the RSC1 (Panel A) and RSC2 (Panel B) groups. Areas in black reflect ventricles. The numbers in Panels A and B indicate the distance (in millimeters) from bregma adapted from *The Rat Brain in Stereotaxic Coordinates* (5th ed.; pp. 49–99), by G. Paxinos & C. Watson, 2005, New York, NY: Academic Press. Copyright 2005 by Elsevier Academic Press. Adapted (or reprinted) with permission. Panel C: Photomicrographs of a coronal section immunostained for NeuN of a representative retrosplenial cortex lesion and a sham control. The scale bar in Panel C represents 200 μm. cb = cingulum bundle; cc = corpus callosum.

In the RSC1 cohort, three rats were excluded due to sparing of the retrosplenial cortex or due to bilateral damage to the most dorsal hippocampus, leaving 13 rats in the RSC1 lesion group and 12 corresponding shams (Sham1). In the RSC1 group, extensive cell loss and gliosis was seen throughout the retrosplenial cortex, in both the granular and dysgranular subregions. Three animals had restricted damage or gliosis in the most dorsal medial tip of the CA1 subfield of the hippocampus (two unilateral). In the remaining case, the bilateral CA1 damage was very restricted. Over all cases, the maximum anterior−posterior extent of dorsal hippocampal damage was 600 μm. Seven animals, including the three with CA1 damage, had slight unilateral thinning of the medial blade of the dentate gyrus just caudal to the splenium. Nine animals had partial sparing of Rga, particularly at its caudal limit. Four rats also had some limited sparing of Rgb (see Figure 3). One rat had slight damage to the anterior cingulate cortex at the junction with retrosplenial cortex, while two showed limited unilateral damage to the secondary motor cortex, lateral to the retrosplenial cortex. A restricted area of gliosis was observed at the junction of the anterior medial and anterior ventral nuclei, as is consistently observed after extensive retrosplenial lesions (Gonzalez, Whishaw, & Kolb, 2003; Neave, Lloyd, Sahgal, & Aggleton, 1994; Vann, Wilton, Muir, & Aggleton, 2003).[Fig-anchor fig3]

In the RSC2 cohort, four rats were excluded due to the presence of bilateral damage to the dorsal hippocampus, leaving 12 lesion animals and 12 corresponding sham-operated animals. In all remaining cases, there was extensive bilateral damage, with marked cell loss and gliosis throughout the entire RSC rostral to the splenium. Caudal to the splenium, eight animals had partial sparing of Rga. Additional cell loss occurred in the most dorsal aspect of the CA1 in the septal hippocampus (three cases bilateral and five unilateral), but the maximum extent of anterior−posterior hippocampal damage was again approximately 600 μm. In five animals (two unilateral and three bilateral) there was also slight thinning of the medial blade of the dentate gyrus caudal to the splenium. In one case there was unilateral damage to the postsubiculum.

### Behavioral Results

#### Experiment 1

##### Acquisition (A+, B+)

The rats learned to lever-press to the A+ and B+ stimuli, as shown by a significant main effect of day, *F*(3, 69) = 25.2, *p* < .001. There was no main effect of lesion or Lesion × Day interaction (both *F*s < 1). By Day 4, no differences were found between the number of lever presses made to each stimulus (*F* < 1), and there was no Lesion × Stimulus interaction (*F* < 1). Rats learned to respond to both stimuli at the same rate, *F*(3, 66) = 1.22, *p* > .05.

##### Negative patterning training (A+, B+, AB−)

Figure 2A displays a difference score calculated by subtracting responding to the compound stimuli (AB−) from the elemental (A+/B+; averaged across the two elemental stimuli) stimuli and across the eight training blocks (three sessions per block). A score above 0 indicates greater responding to the reinforced stimuli (i.e., A+/B+) relative to the nonreinforced compound (AB−). Inspection of this figure reveals that both groups gradually solved the negative patterning problem but that the RSC1 group appeared slower to learn to discriminate between the elemental and compound stimuli. ANOVA revealed a main effect block, *F*(7, 161) = 52.43_,_ *p* < .001. Despite the impression from Figure 2A, there were no statistically significant differences between the RSC1 and Sham1 groups in their ability to solve the negative patterning problem, nor was there any statistical evidence that the RSC1 group acquired the discrimination more slowly. There was no effect of lesion, *F*(1_,_23) = 1.7_,_ *p* = .2, or a Block × Lesion interaction, *F*(7, 161) = 1.7, *p* = .11. Furthermore, one-sample *t* tests confirmed that both Sham1, *t*(11) = 4.1, *p* < .012, and RSC1 animals, *t*(12) = 2.9, *p* < .05, discriminated between the elemental and compound stimuli (i.e., a difference score above 0) from the same block of sessions (Block 5). Final performance of the two groups is displayed in Figure 2B. As is clear from Figure 2B, the overall level of responding and the magnitude of the discrimination were equivalent in the two groups. ANOVA yielded a main effect of stimulus type, *F*(1, 23) = 59.94_,_ *p* < .001, but no Lesion × Stimulus Type interaction (*F* < 1) or a main effect of lesion (*F* < 1).

Likewise there were no differences in performance between the two groups during the 50-s period of stimulus presentation during which correct lever presses were reinforced (see Table 1). ANOVA revealed a main effect of stimulus type, *F*(1, 23) = 169.8_,_ *p* < .001, and a Block × Stimulus Type interaction, *F*(2.6,59.5) = 68.6_,_ *p* < .001, but no main effect or interactions involving lesion, maximum *F*(1, 23) = 2.68_,_ *p* = .12. Unsurprisingly, magazine activity was higher during reinforced than nonreinforced trials, because animals retrieved rewards following reinforced lever presses. ANOVA yielded a main effect of stimulus type, *F*(1, 23) = 182.8, *p* < .001, and a Block × Stimulus Type interaction, *F*(4.3, 98.8) = 15.5_,_ *p* < .001, but no effects or interactions involving lesion, maximum *F*(3.01, 58.5) = 2.61_,_ *p* = .06.[Table-anchor tbl1]

##### Acquisition of C+ and negative patterning with C+ (A+, B+, C+, AB−)

Both groups readily acquired lever-pressing to the C+ stimulus. Mean lever presses per minute (±*SEM*) were 31.9 (±4.2) for Sham1 and 38.7 (±3.9) for RSC1 across the three sessions. There was no difference in the number of lever presses made by each group, *F*(1, 23) = 1.36, *p* = .26. There was no main effect of day on lever presses per minute, presumably due to ceiling effects, and there was no Day × Lesion interaction (both *F*s < 1). During the next four sessions the animals discriminated between the three rewarded elemental stimuli (A+, B+, C+) and the nonrewarded compound (AB−). Both groups continued to solve the negative patterning problem. There was a main effect of stimulus, *F*(1, 23) = 38.1, *p* < .01, and session, *F*(3, 69) = 9.7_,_ *p* < .001, and a Session × Stimulus interaction, *F*(3, 69) = 9.1_,_ *p* < .01. There was no effect of lesion, *F*(1, 23) = 1.54, *p* = .23, or any interactions involving lesion group, maximum *F*(3, 69) = 1.17, *p* = .33. On the final day, mean lever presses per minute (±*SEM*) during presentation of the rewarded elemental stimuli (A+, B+, C+) were 30.1 (±3.3) for Sham1 and 33.6 (±3.4) for RSC1, and during presentation of the compound stimuli (AB−), 17.7 (±3.3) for Sham1 and 24.6 (±3.2) for RSC1.

##### Transfer Test 1 (A+ B+, AB−, C+, AC−, BC−)

**Figure 4 fig4:**
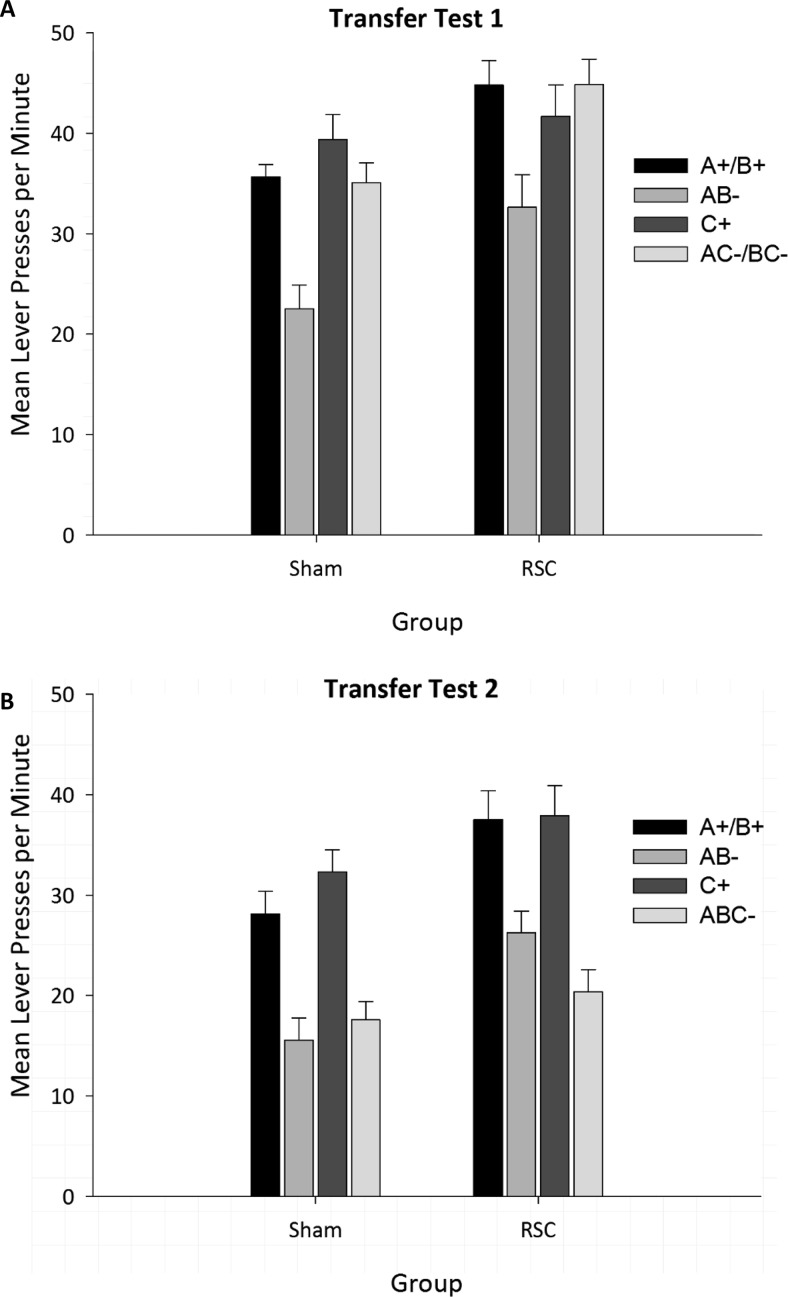
Performance of Sham1 and RSC1 lesion animals during the negative patterning transfer tests. The figure depicts mean lever presses per minute during the first 10 s of stimulus presentation (no reward available). The lack of suppression in responding to the compounds AC− and BC− (Transfer Test 1) demonstrates that neither group had acquired the negative patterning discrimination by using nonconfigural cues such as stimulus intensity or numerosity. Transfer Test 2 confirms that the animals were able to suppress responding during presentation of compounds involving C (magazine light), because responding to ABC− was lower than to C+. Error bars indicated the standard error of the mean. A represents tone stimulus, and B represents flashing panel light stimulus. RSC = retrosplenial cortex lesion.

To determine whether the rats might have solved the negative patterning discrimination by reference to the number of stimuli presented, a transfer test was carried out using novel compounds involving the C stimulus. For analysis, a mean of lever-press activity was taken across the original elemental stimuli (A+/B+) and the compounds containing the magazine light (AC−/BC−). As is clear from Figure 4, performance on this transfer test did not differ by lesion group: There was no significant main effect of lesion (*F* < 1) or Stimulus × Lesion interaction, *F*(1, 23) = 1.44, *p* = .24. However, there was a main effect of stimulus, *F*(2.3, 55.1) = 16.58, *p* < .001. Critically, pairwise comparisons (Bonferroni-corrected) showed that responding to AB− was lower than to any of the other stimuli (in all comparisons, *p* < .001). Furthermore, there were no differences between any of the other stimuli (all *p*s > .05), demonstrating that animals had not learned to withhold responding to the AB− compound based on simply the number of elements present.[Fig-anchor fig4]

##### Transfer Test 2 (A+, B+, AB−, C+, ABC−)

To determine whether rats were able to suppress responding to compound stimuli containing the C stimulus, a final transfer test involving the compound ABC− was completed. As is clear from Figure 4, both groups of animals were able to suppress responding to the ABC− compound. ANOVA yielded an effect of stimulus, *F*(3, 69) = 31.46, *p* < .001, but no effect of lesion, *F*(1, 23) = 1.58, *p* = .22, or any interaction between these factors, *F*(3, 69) = 1.51, *p* = .22. Pairwise comparisons confirmed that responding to ABC− was lower than to all elemental stimuli (all *p*s < .001) but did not differ from responding to AB− (*p* > .05). Similarly, responding to the nonrewarded compound AB− was lower than to the rewarded elemental stimuli (all *p*s < .001).

#### Experiment 2

##### Acquisition of conditioned inhibition

**Figure 5 fig5:**
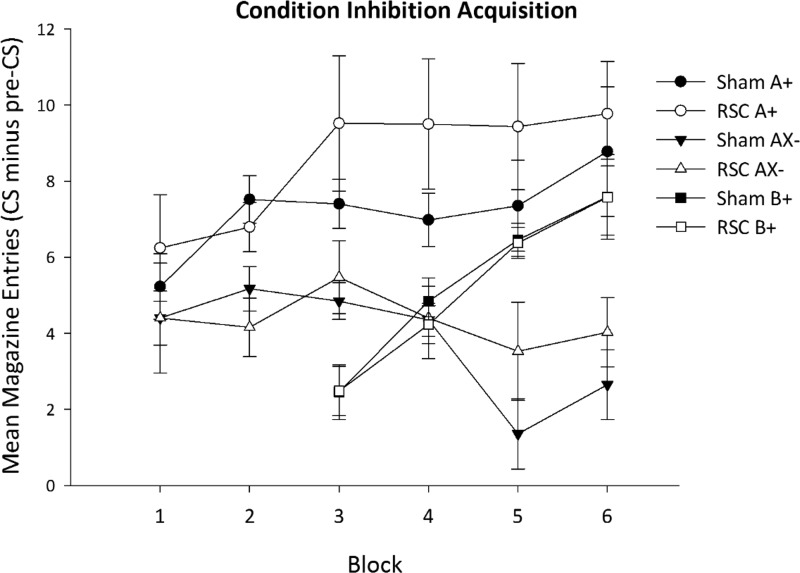
Acquisition of conditioned inhibition in the Sham2 and RSC2 groups. The figure depicts mean magazine entries during the 10-s stimulus presentation minus the number of entries during the 10-s period preceding the onset of the conditioned stimulus (CS). Food was delivered after the offset of A (tone) and B (flashing panel light), but food was omitted on AX− trials when the tone was presented in compound with X (house light extinguished). Error bars indicated the standard error of the mean. RSC = retrosplenial cortex lesion.

By the end of the six blocks of acquisition, responding during A+ trials was higher than to AX− (see Figure 5). ANOVA confirmed an effect of stimulus, *F*(1, 22) = 20.3, *p* < .001, and a Block × Stimulus interaction, *F*(5, 110) = 7.1, *p* < .001. As is clear from Figure 5, acquisition of the discrimination was not affected by lesion group. ANOVA yielded no effect of lesion or any interactions involving lesion group (all *F*s < 1). In Blocks 3–6, responding to B+ increased in both groups, *F*(3, 66) = 25.3, *p* < .001, but again this increase was unaffected by lesion group (*F* < 1).[Fig-anchor fig5]

To compare responding during the three stimuli over the final training block, pairwise comparisons (Bonferroni-corrected) were carried out on the data. There was no significant difference between responding during A+ and B+ trials in either the Sham2 (*p* = .14) or lesion (*p* = .35) animals. However, responding during both A+ and B+ trials was higher relative to AX– trials in both groups (minimum *p* < .05), providing evidence for acquisition of inhibitory associations between X and reward.

##### Summation test

**Figure 6 fig6:**
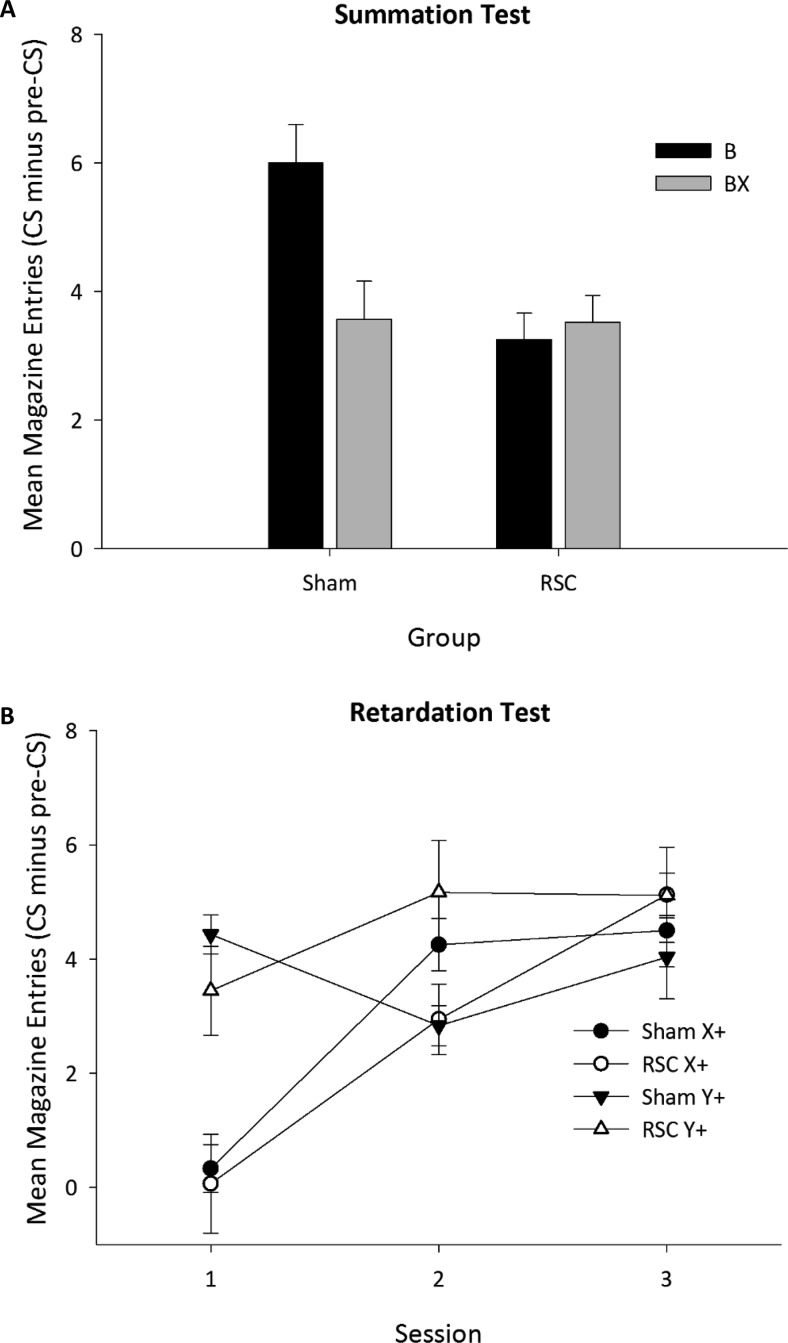
Panel A: Performance of the Sham2 and RSC2 groups during the summation test. In extinction, animals received B (flashing panel light) only trials as well as trials in which B was presented in compound with the conditioned inhibitor X (house light extinguished) for the first time. The figure depicts mean magazine entries during the 10-s stimulus presentation minus the number of entries during the 10-s period preceding the onset of the conditioned stimulus (CS). Panel B: Performance of the Sham2 and RSC2 groups during the retardation test. The acquisition of an excitatory conditioned response to the conditioned inhibitor X (house light off) was compared relative to the novel stimulus Y (magazine light). The figure depicts mean magazine entries during the 10-s stimulus presentation minus the number of entries during the 10-s period preceding the onset of the CS, across the three sessions. Error bars indicate the standard error of the mean. RSC = retrosplenial cortex lesion.

Figure 6A shows responding to B and BX during the summation test. In Sham2 animals, presenting the putative conditioned inhibitor (X) simultaneously with the conditioned excitor (B) reduced responding compared with presentation of the conditioned excitor alone. In contrast in the RSC2 group, inhibition accrued to X did not transfer to BX, because responding was equivalent during B and BX presentations. ANOVA confirmed this description of the data: There was no effect of stimulus, *F*(1, 22) = 2.96, *p* = .1, or lesion (*F* < 1), but critically there was an interaction between stimulus and lesion group, *F*(1, 22) = 4.61, *p* < .05. Simple effects analysis of this interaction confirmed that in Sham2 animals the magnitude of responding during B trials was greater than during BX trials, *F*(1, 22) = 7.47, *p* < .05, but there was no differential rate of responding in the lesion animals (*F* < 1).[Fig-anchor fig6]

##### Retardation test

Following the summation test, the animals underwent six further sessions of training on the original A+ AX− discrimination. Both groups continued to respond more during A+ trials than during AX− trials, *F*(1, 22) = 26.1, *p* < .001, with no effects of block or lesion or any interactions, maximum *F*(1, 22) = 1.67, *p* = .21. In the final training block, rats were presented with a novel, unrewarded stimulus Y. There was no difference between the groups in the rate of responding to the novel Y stimulus (*F* < 1). Pairwise comparisons were carried out on the mean responding during the three stimuli (A+, AX−, and Y−) over the last block of two sessions. In both groups, responding during A+ trials was higher than during AX− (*p* < .01) and Y− (*p* < .05) trials, but responding during Y− and AX− did not differ (minimum *p* = .35). Mean response rates (±*SEM*) were Sham2: A+ = 6.86 (±2.5), AX− = 2.08 (±1.1), Y− = 2.88 (±.7), and RSC2: A+ = 11.81 (±2.8), AX− = 2.88 (±1.3), Y− = 2.77 (±.8).

Figure 6B shows mean responding during presentation of X (previously trained as a conditioned inhibitor) and Y (a habituated novel stimulus) across the three sessions of the retardation test. Both groups showed a clear retardation effect (i.e., lower responding to X than to Y) because X was slower to acquire excitatory properties relative to the neutral stimulus Y, thereby indicating that it had acquired inhibitor properties during Stage 1. ANOVA revealed a main effect of stimulus, *F*(1, 22) = 11.18, *p* < .01; a main effect of session, *F*(2, 44) = 20.91, *p* < .001; and a Stimulus × Session interaction, *F*(2, 44) = 11.71 *p* < .001, but no Lesion × Stimulus interaction, *F*(1, 22) = 2.1, *p* = .16. There was, however, a three-way Lesion × Stimulus × Session interaction, *F*(2, 44) = 3.27, *p* < .05. Simple effects confirmed that this interaction arose due to differences in conditioning to Y (Session × Lesion interaction), *F*(2, 44) = 4.03, *p* < .05, rather than X (no Session × Lesion interaction), *F*(2, 44) = 1.23, *p* = .3, indicating that the retardation effect (i.e., retarded acquisition of excitatory responding to X) was equivalent in both groups of animals. Furthermore, by the end of Session 3 there was no difference in responding to either X or Y in either group (all *F*s < 1).

## Discussion

The current set of experiments examined the role of the retrosplenial cortex in the processing of nonspatial stimuli with the aim of contrasting different theoretical accounts of retrosplenial cortex function. In Experiment 1, animals with excitotoxic lesions in the retrosplenial cortex were tested on a negative patterning discrimination that taxes the ability to bind together environmental stimuli into a unique (configural) representation. The retrosplenial cortex lesion group successfully acquired the discrimination and reached the same level of performance as their surgical controls by the end of training. Follow-up tests confirmed that the retrosplenial cortex group had not solved the discrimination by nonconfigural means such as numerosity or intensity of the stimuli. These results suggest that in the nonspatial domain, at least, the RSC is not required for the binding together of environmental stimuli into unique representations. Furthermore, Experiment 2 found no evidence that retrosplenial cortex lesions disrupt the acquisition of conditioned inhibition, a task that also requires animals to learn interrelationships between stimuli. Experiment 2 did, however, reveal specific conditions under which the RSC may be involved in the processing of nonspatial information.

The finding here that excitoxic lesions in the retrosplenial cortex did not impair configural learning or the acquisition of conditioned inhibition is notable because these results stand in contrast to results in other reports of impaired processing of environmental stimuli of the nature employed here with analogous response requirements. It should also be noted that although the retrosplenial lesions in Experiment 1 did not disrupt configural learning, these same animals were impaired on tests of cross-modal recognition and spatial memory (Hindley et al., 2014; Nelson, Hindley, Pearce, Vann, & Aggleton, 2015). Thus, the intact performance of these animals on tests of configural learning is unlikely to be due to the ineffectiveness of the lesion or tissue sparing. Indeed, the lesions in the current cohort involved tissue along almost the entire anterior−posterior axis of the retrosplenial cortex, which is significant because more restricted lesions have the potential to produce null results (Aggleton & Vann, 2004; Neave et al., 1994). Furthermore, although the lesions in Experiment 2 did not affect the acquisition of conditioned inhibition per se, the lesions did disrupt performance on the summation test. One obvious explanation of differences between the current findings and previous findings is methodological, because the current results were obtained with excitotoxic lesions rather than mechanical lesions that can destroy fibers of passage and potentially exacerbate the functional impact of the damage (Keene & Bucci, 2008a; Robinson et al., 2011). Moreover, in a separate set of studies we have also shown that excitotoxic lesions in the retrosplenial cortex spare performance on tests of intra- and extradimensional set-shifting, which require animals to track the behavioral relevance of multiple multidimensional stimuli (Powell et al., 2017).

This pattern of null results on tests of nonspatial stimuli is problematic for theories of RSC function that posit a fundamental role for this cortical structure in stimulus integration (Todd & Bucci, 2015). According to such theories, the retrosplenial cortex acts as a site of integration of incoming environmental stimuli, binding such stimuli together to form conjunctive representations, a function that potentially underlies RSC involvement in processing spatial and contextual information. These questions around the precise role that the retrosplenial cortex plays in processing spatial and nonspatial stimuli memory mirror to some extent historical debates about the mnemonic functions of the hippocampus, for example, configural theories of hippocampal function (Rudy & Sutherland, 1995). It is of particular note that retrosplenial lesions spared performance on the negative patterning task because it is a form of configural learning that can be solved only by learning the conjunction of cues rather than just the individual stimulus elements. The task, therefore, provides a direct test of the ability of animals with retrosplenial cortex lesions to bind together multimodal sensory information into a unique representation. Thus, as is the case with the hippocampus (Bussey et al., 2000; Davidson, McKernan, & Jarrard, 1993; Gallagher & Holland, 1992), retrosplenial lesions that reliably disrupt spatial (Mitchell, Czajkowski, Zhang, Jeffery, & Nelson, 2018) and contextual (Keene & Bucci, 2008b; Todd et al., 2017) learning are without effect on a nonspatial test of configural learning. One factor that may be crucial is that the negative patterning task is acquired incrementally over multiple sessions, whereas spatial or contextual learning often involves rapid one-trial learning in which a “snapshot” of the conjunction of cues is formed. Furthermore, the response requirements of these tasks are strikingly different.

Experiment 2 provided a further test of the ability of retrosplenial lesion animals to learn the interrelationship between stimuli. Despite previous evidence that retrosplenial cortex is required for cue selection (Keene & Bucci, 2008a; Robinson et al., 2011), in our hands, excitotoxic retrosplenial lesions did not impair the acquisition of conditioned inhibition. However, Experiment 2 did reveal a selective lesion deficit on the summation test, in which the putative inhibitor is paired with a novel excitatory stimulus, thereby requiring the animals to generalize what had been learned about that stimulus to a novel situation. Intact animals readily suppress responding when the conditioned inhibitor is paired with the novel stimulus, but this effect was absent in the lesion group. Given that the same animals had successfully acquired conditioned inhibition in the previous stage, it is highly unlikely that a general inability to inhibit responding can explain this pattern of results. Perhaps more important, the summation test occurred in the absence of reinforcement, requiring the animals to transfer what had previously been learned to a novel situation without the ability to directly experience stimulus contingencies. Consistent with this proposal, the same retrosplenial lesion group passed the retardation test, that is, acquired an excitatory conditioned response to the conditioned inhibitor when it was subsequently paired with reinforcement. Crucially, the retardation test was reinforced, thereby allowing the animals to directly experience “online” the change in reward contingencies without the need to rely on previously acquired stimulus representations. These findings mirror evidence from sensory preconditioning procedures in which animals also have to update responding to a stimulus in the absence of reinforcement (Robinson et al., 2014).

On the basis of the current results, it would seem likely that the role of the retrosplenial cortex in processing nonspatial stimuli is either highly selective or shared with other sites. The demonstration here that excitotoxic retrosplenial lesion animals are able to acquire a negative patterning discrimination and conditioned inhibition, combined with previous evidence that the retrosplenial cortex lesions do not disrupt second-order conditioning (Todd et al., 2016) or attentional set-shifting (Powell et al., 2017), suggests that stimulus integration is unlikely to be an overarching property of retrosplenial function. Rather, lesion deficits emerge on those tasks where there is a mismatch between previously acquired representations and current contingencies or stimulus relationships. In particular, impairments are observed when animals are tested in the absence of reinforcement and, consequently, are unable to experience online reward contingencies and stimulus interrelationships. or acquire new learning (Cowansage, 2018; Hindley et al., 2014; Nelson et al., 2014; Powell et al., 2017; Robinson et al., 2014). This proposition needs further empirical testing with chemogenetic or optogenetic approaches that allow far greater temporal precision than is possible with conventional lesion studies. Nevertheless, this suggestion fits with proposals that the retrosplenial cortex serves as a comparator that translates representations from one frame of reference to another (Burgess, 2002; Byrne et al., 2007; Vann et al., 2009).

## Figures and Tables

**Table 1 tbl1:** Acquisition of Negative Patterning Discrimination for Sham1 and RSC1 Lesion Animals in Three-Session Blocks (Experiment 1)

Subject and stimulus	Block							
	1	2	3	4	5	6	7	8
Sham1								
A+/B+	26.4 (±2.1)	28.9 (±2.5)	32.7 (±2.1)	32.2 (±2.4)	30.9 (±2.1)	30.2 (±2.4)	31.7 (±2.5)	29.8 (±2.7)
AB−	30.2 (±2.1)	29.4 (±2.3)	28.2 (±2.5)	23.0 (±2.9)	19.4 (±2.7)	16.5 (±2.9)	18.3 (±2.9)	14.4 (±2.6)
RSC1								
A+/B+	23.6 (±3.1)	25.1 (±3.1)	26.8 (±3.4)	27.5 (±3.5)	27.0 (±3.5)	27.4 (±3.5)	29.0 (±3.6)	27.7 (±2.9)
AB−	26.7 (±2.6)	24.3 (±3.0)	23.1 (±2.1)	17.1 (±3.4)	15.2 (±3.8)	13.2 (±3.4)	14.0 (±3.5)	11.8 (±3.5)
*Note.* Data represent mean lever presses during the 50 s of stimulus presentation in which correct lever presses were rewarded, that is, during presentation of the reinforced tone (A+) and flashing panel light (B+) stimuli, as well the mean lever presses during the nonreinforced compound presentation of these stimuli (the standard error of the mean appears in parentheses). RSC = retrosplenial cortex lesion.								

**Figure 1 fig1:**
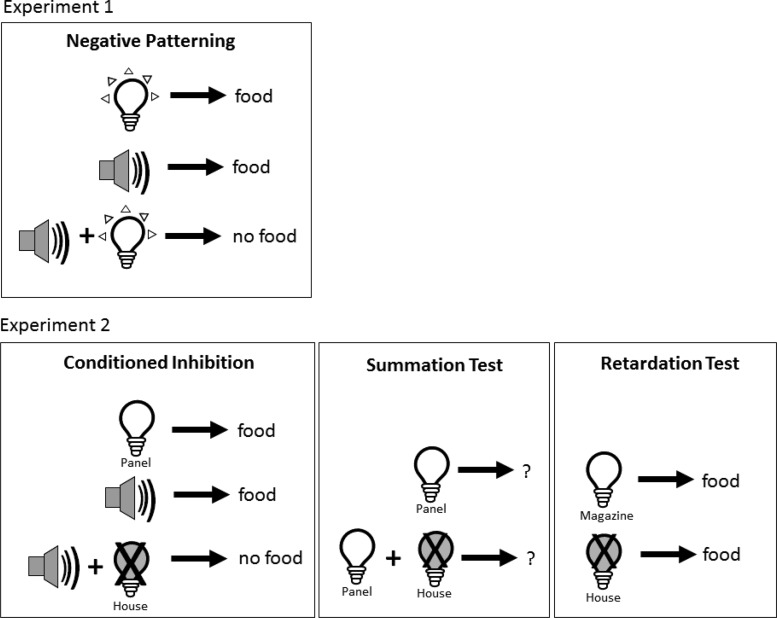
A schematic diagram of the experimental design for Experiments 1 and 2. In Experiment 1, animals acquired a negative patterning discrimination in which a tone and a flashing panel light were rewarded when presented alone but were unrewarded when presented in compound. In Experiment 2, food was presented after a panel light and a tone, but food was omitted when the tone was presented in combination with the house light extinguished (conditioned inhibitor). The animals subsequently underwent summation and retardation tests. The summation test examined whether the putative conditioned inhibitor would function as a conditioned inhibitor when presented with a different excitatory stimulus (panel light). Consequently, animals received trials in which the panel light was presented alone or in combination with the conditioned inhibitor (house light extinguished). Finally, in the retardation test the conditioned inhibitor was repeatedly paired with food, and responding to the conditioned inhibitor was compared with responding to a novel stimulus (magazine light) that was also paired with food. If the stimulus had acquired inhibitory properties, acquisition of an excitatory response should be impaired (retarded) relative to the novel stimulus.
